# DNA barcoding and gene expression recording reveal the presence of cancer cells with unique properties during tumor progression

**DOI:** 10.1007/s00018-022-04640-4

**Published:** 2022-12-23

**Authors:** Yuka Umeki, Noriaki Ogawa, Yuko Uegaki, Kotaro Saga, Yasufumi Kaneda, Keisuke Nimura

**Affiliations:** Division of Gene Therapy Science, Department of Genome Biology, Graduate School of Medicine, Osaka University, 2-2 Yamada-Oka, Suita, Osaka 565-0871 Japan

**Keywords:** DNA barcode, Gene expression recording, Cancer stem cell, stgRNA, NANOG, POU5F1

## Abstract

Tumors comprise diverse cancer cell populations with specific capabilities for adaptation to the tumor microenvironment, resistance to anticancer treatments, and metastatic dissemination. However, whether these populations are pre-existing in cancer cells or stochastically appear during tumor growth remains unclear. Here, we show the heterogeneous behaviors of cancer cells regarding response to anticancer drug treatments, formation of lung metastases, and expression of transcription factors related to cancer stem-like cells using a DNA barcoding and gene expression recording system. B16F10 cells maintained clonal diversity after treatment with HVJ-E, a UV-irradiated Sendai virus, and the anticancer drug dacarbazine. PBS treatment of the primary tumor and intravenous injection of B16F10 cells resulted in metastases formed from clones of multiple cell lineages. Conversely, BL6 and 4T1 cells developed spontaneous lung metastases by a small number of clones. Notably, an identical clone of 4T1 cells developed lung metastases in different mice, suggesting the existence of cells with high metastatic potential. Cas9-based transcription recording analysis in a human prostate cancer cell line revealed that specific cells express POU5F1 in response to an anticancer drug and sphere formation. Our findings provide insights into the diversity of cancer cells during tumor progression.

**Supplementary Information:**

The online version contains supplementary material available at 10.1007/s00018-022-04640-4.

## Introduction

Cancer cell proliferation promotes tumor growth with continuously increasing diversity in gene expression profiles, resulting in tumor heterogeneity that allows tumors to include various clonal proportions adapting to the tumor microenvironment, resistance to anticancer treatments, and migration to distant metastatic lesions [3, 37]. Recent large-scale cancer genome analysis has revealed a hierarchical evolution in cancer progression and identified unique mutations in distant metastases from a primary tumor, suggesting that metastatic cancer cells acquire de novo mutations during migration or proliferation in the metastases [7, 39]. However, genetic mutations are not the only cause of the diverse phenotypes of cancer cells, but epigenetic regulation also contributes to the development of tumor diversity [6, 32].

In cancer progression, resistance to anticancer therapies and metastatic potential are the major factors contributing to cancer-related deaths [18]. Thus, it is critical to identify whether cancer cells include a pre-existing population with resistance to anticancer therapies and metastatic abilities or whether these cancer cells appear during cancer progression [9, 10]. Cancer stem-like cells (CSCs) are thought to initiate tumorigenesis and resist environmental stress to promote cancer progression [1]. Pluripotent transcription factors, including NANOG, SOX2, and OCT3/4, contribute to the establishment of stem cell-like properties in cancer cells, similar to embryonic stem cells [28]. Nonetheless, whether CSCs constitute as the major cell type in tumors and survive anticancer drug treatment remains unclear.

DNA barcoding systems, using random DNA sequences as barcodes, have identified cell proliferation and migration dynamics, including hematopoiesis progression [21, 36] and distinct populations in 4T1 murine breast cancer cell lines that contribute to metastasis [40]. The application of a DNA barcoding system to patient-derived xenografts (PDXs) and mouse tumor models has revealed diverse clonal proliferation patterns in primary tumors and metastases in mice, clonal responses to anticancer drugs, and diverse tumor-initiating abilities [19, 24, 25]. Some cancer cell lines indicate the existence of elite cells that initiate tumorigenesis, generate metastases, and survive anticancer drug treatment [2, 19, 25, 29]. However, anticancer drugs do not always select specific clonal populations [24]. Moreover, the contribution of rare clonal proportions with distinct capabilities, such as CSCs, to cancer progression is unclear.

We examined whether a cancer cell population with the distinct capabilities of proliferation, metastasis, and resistance to anticancer drugs is pre-existing or develops post treatment in multiple cancer cell lines using DNA barcoding and gene expression recording systems. To this end, we utilized mouse melanoma cells B16F10 and B16/BL6 (BL6) and the mouse mammary carcinoma cell line 4T1 to identify the distinct cancer cell population promoting cancer progression. These cell lines are often used to analyze metastasis and the human prostate cancer cell line DU145 for tracing CSCs under environmental stress. We previously found that DU145 cells contain NANOG-positive CSCs that escape NK cell attack [13, 31]. In this study, we used a lentivirus-based DNA barcoding system to distinguish cancer cells during cancer progression. We also established DU145 with the insertion of Cas9 immediately before the stop codons of the *NANOG*, *SOX2*, and *POU5F1* genes. The expression of these transcription factors in the self-targeting guide RNA (stgRNA) was recorded as the frequency of mutations in the sequence, reflecting Cas9 activity [27]. Our results revealed multiple clones and a few clone-composed metastases, the stochastic appearance of anticancer drug-resistant cells, and POU5F1 expression in specific cells responding to either sphere formation or anticancer drugs. Thus, therapeutic strategies are required to target pre-existing and post-treatment cancer cells with metastatic and anticancer drug resistance capabilities.

## Results

**Fig. 1:**
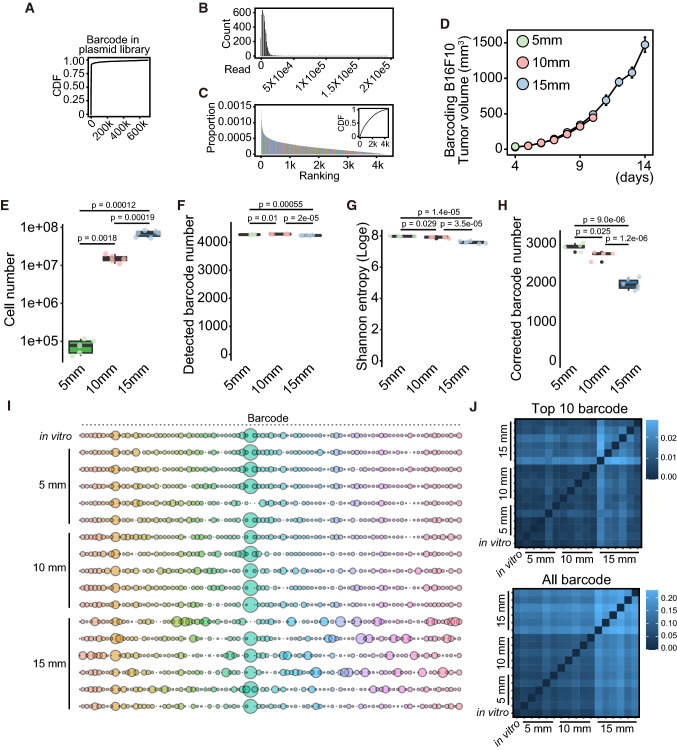
The switch from homogeneous to heterogeneous cancer cell proliferation during tumor growth in DNA-barcoded B16F10 cells. **A** Cumulative distribution fraction (CDF) of barcodes in plasmid library. *X*-axis represents the number of barcodes. **B** Histogram of barcodes commonly detected in the plasmid and cells. *X*-axis represents the number of reads, whereas *Y*-axis represents the number of barcodes. Bin size is 1000. **C** Histogram and CDF of a proportion of barcodes commonly detected in the plasmid and cells. *X*-axis represents the ranking of barcode in the plasmid, whereas Y-axis represents the proportion of barcode. **D** The tumor growth curve of barcoding B16F10 cells in C57BL/6N. The tumors with 5, 10, and 15 mm diameters were collected at the indicated days. The day was counted from the inoculation of cancer cells (*n* = 5 for 5 and 10 mm diameter tumors and *n* = 6 for 15 mm diameter tumors). **E**–**H** Box plots of cell number collected from tumors (**E**), detected barcode number (**F**), Shannon entropy (Loge) (**G**), and corrected barcode number (**H**). *p* value was calculated by Welch’s *t*-test with multiple comparisons Bonferroni correction. **I** Bubble plot of top 10 barcodes from each of the 17 samples (*n* = 1 for in vitro, *n* = 5 for 5 mm, *n* = 5 for 10 mm, and *n* = 6 for 15 mm). The total bubble number is 93 in each line since we aggregated the duplicated barcodes detected in the top 10 barcodes of different samples. Bubble size indicates the proportion of the barcode. **J** Heatmap of Jensen–Shannon divergence calculated using top 10 barcodes in each sample or all barcodes

DNA barcoding system identifies each cell, allowing analysis of cell lineage, proportion, and dynamics at a single-cell level. We designed a lentivirus plasmid with random sequences comprising 30 bases each. The plasmids included 700,807 variations in the depth of ~ 20 M reads, suggesting that 7.1% variation in barcode occupied 95% of the library (Fig. 1A). Sequencing error contributes to a nucleotide mismatch, appearing as hamming distance of 1–2. We sought to reduce the bias causing sequencing errors by aggregating barcodes according to Hamming distance and selecting barcodes detected in plasmids and all barcoded-B16F10, BL6, and 4T1 cells (see Materials and Methods). The obtained 4,367 barcodes did not contain barcodes with excessive amounts (Fig. 1B) and showed an average abundance (Fig. 1C). Thus, we analyzed the dynamics of these 4367 barcodes during cancer progression in B16F10, BL6, and 4T1 cells.

First, we determined whether cancer cells show diversity in cell proliferation in vivo. We inoculated B16F10 cells on the backs of C57BL/6N mice and collected tumors with diameters 5, 10, and 15 mm, corresponding to 4, 10, and 14 days after inoculation (Fig. 1D). Cell numbers increased ~ 200-fold from 5 to 10 mm tumors and ~ fivefold from 10 to 15 mm tumors (*p* = 0.0018 and *p* = 0.00019, respectively; Fig. 1E). We found a significant difference in the detected barcode numbers among the samples, although the difference was slight (Fig. 1F). Because the barcode data were skewed, we calculated the corrected barcode number using Shannon entropy to obtain the effective barcode numbers by removing skewing from the data [38] (Fig. 1G and H). The original barcoded-B16F10 cells contained ~ 3250 barcodes. B16F10 tumors drastically increased in cell numbers from 5 to 10 mm tumors, and the number of barcoded cells decreased slightly after cell proliferation (2930–2723, *p* = 0.025). In contrast, an ~ fivefold increase in cell number from 10 to 15 mm significantly decreased the number of barcode variations (2723–2000, *p* = 1.2e–06, Fig. 1H). We obtained the top 10 barcodes with the highest number of reads from each sample and created a list of those barcodes. The amounts of barcodes in that list in each sample were used to calculate JS divergence. We then analyzed the change in the abundance of individual barcodes of the top 10 in each sample. Results suggested that the composition of barcodes significantly changed during tumor growth from 10 to 15 mm but not during tumor growth from 5 to 10 mm compared to in vitro barcoded-B16F10 cells (Fig. 1I). We then applied Jensen–Shannon (JS) divergence to compare the similarities between samples [20]. A high JS divergence score indicates low similarity between samples, whereas a low score indicates high similarity. We detected high similarity in the composition of barcodes among in vitro B16F10 cells (5 and 10 mm tumors). In comparison, 15 mm tumors showed different compositions from other samples based on the top 10 barcodes from each sample or all barcodes (Fig. 1J). These data indicate the transition of cell proliferation profiles from homogeneous to heterogeneous during tumor growth in B16F10 tumors.

**Fig. 2:**
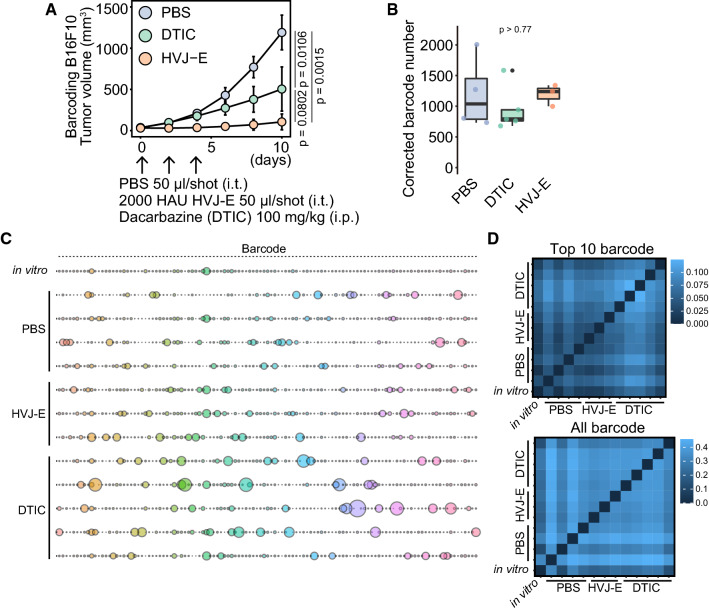
Stochastic resistance to HVJ-E and DTIC in barcoded B16F10 tumors. **A** The tumor growth curve of barcoding B16F10 cells in C57BL/6N. HVJ-E (2000 HAU) and PBS were intratumorally injected into the tumors on days 0, 2, and 4. DTIC (100 mg/kg) was intraperitoneally injected on days 0, 2, and 4. *p* value was calculated by Welch’s *t*-test with multiple comparisons Bonferroni correction (*n* = 4 for PBS, *n* = 3 for HVJ-E, and *n* = 5 for DTIC). **B** Box plot of the corrected barcode number by Shannon entropy. *P* value was calculated by Welch’s *t*-test with multiple comparisons Bonferroni correction. **C** The bubble plot of top 10 barcodes from each of the 13 samples (*n* = 1 for in vitro, *n* = 4 for PBS, *n* = 3 for HVJ-E, and *n* = 5 for DTIC). The total bubble number is 117 in each line since there is a redundancy. Bubble size indicates the proportion of the barcode. **D** The heatmap of JS divergence calculated using top 10 barcodes in each sample or all barcodes

Next, we examined whether the repression of tumor growth by anticancer drugs decreases the variety of barcodes and enriches a specific clone in B16F10 tumors. We analyzed two anticancer drugs with different molecular mechanisms to repress tumor growth: hemagglutinating virus of Japan envelope (HVJ-E), a UV-irradiated Sendai virus that almost lost replicating ability, and dacarbazine (DTIC) [15, 17, 23]. HVJ-E and DTIC significantly suppressed B16F10 tumor growth (*p* = 0.0015 and *p* = 0.0106, respectively; Fig. 2A). Notably, HVJ-E and DTIC-suppressed tumor growth did not decrease the corrected barcode number in B16F10 tumors, compared to the PBS-treated group (*p* > 0.77, Fig. 2B). We did not find the enrichment of a specific barcode across HVJ-E and DTIC-treated tumors (Fig. 2C). We obtained the top 10 barcodes as described in Fig. 1. DTIC tended to affect the composition of the top 10 barcodes more than PBS and HVJ-E (Fig. 2D). In contrast, the two anticancer drugs were similar to PBS in affecting the composition of all barcodes (Fig. 2D). These results suggest that B16F10 cells do not contain pre-determined anticancer drug-resistant cells and that the cells surviving in the tumor are stochastically determined during tumor growth even under anticancer drug treatment.

**Fig. 3:**
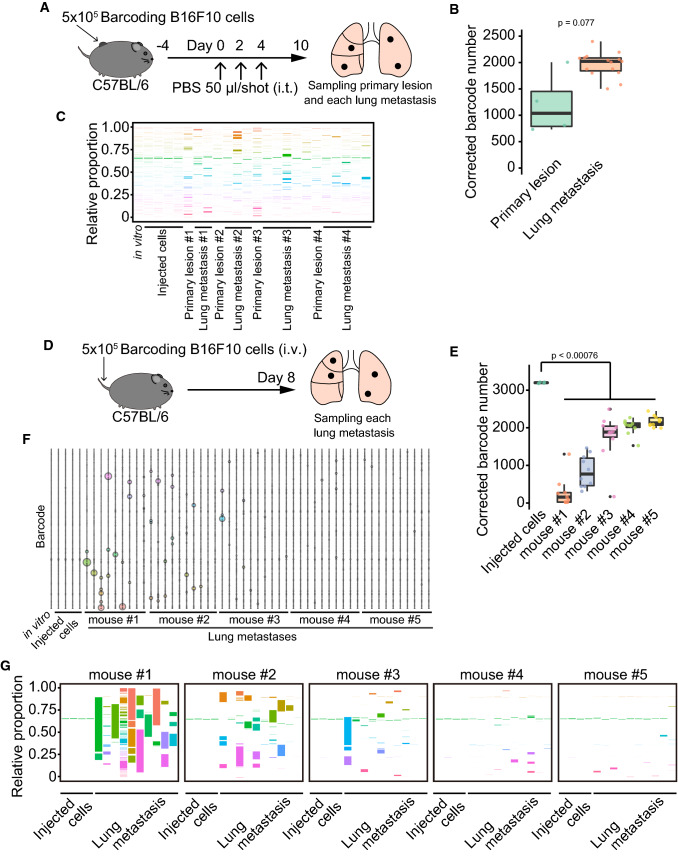
A method-dependent cellular composition of the lung metastases in B16F10 tumors. **A** Schematic diagram depicting the establishment of C57BL/6N mice with lung metastases by three intratumor injections of PBS. The primary tumor and lung metastases were separately collected on day 10 after the first PBS injection. **B** Box plot of the corrected barcode number by Shannon entropy in the PBS-treated mice. PBS sample in Fig. 2B was used for primary lesion data. *p* value was calculated by Welch’s *t*-test. **C** 100% stacked bar plot of the proportions of barcodes in the PBS-treated mice. **D** Schematic diagram illustrating the establishment of C57BL/6N mice with lung metastases by intravenous injection of B16F10 cells. The lung metastases were collected separately on day 8 after the injection. **E** Box plot representing the corrected barcode number by Shannon entropy in the B16F10 cells-intravenously-injected mice. *P* value was calculated by Welch’s *t*-test with multiple comparisons Bonferroni correction. **F** The bubble plot of top 10 barcodes from each of the 54 samples (*n* = 1 for in vitro, *n* = 4 for the injected cells, *n* = 9 for mouse #1, *n* = 10 for mouse #2, *n* = 10 for mouse #3, *n* = 10 for mouse #4, and *n* = 10 for mouse #5) in the B16F10 cells-intravenously-injected mice. The total bubble number is 165 in each line since there is a redundancy. Bubble size indicates the proportion of the barcode. **G** 100% stacked bar plot of the proportions of barcodes in the B16F10 cells intravenously injected mice

Next, we determined the cellular composition of lung metastases comprising B16F10 cells. We created a B16F10 lung metastasis model by injecting PBS into the primary tumor and intravenously injecting B16F10 cells to examine whether these methods affect the cellular composition of lung metastases. Three injections of PBS into B16F10 primary tumors promoted the formation of lung metastasis. We separately collected the primary tumor and metastases and then analyzed the barcode composition (Fig. 3A). The corrected barcode number detected in the lung metastases was comparable to that detected in the primary tumor (*p* = 0.077, Fig. 3B). We also did not find enrichment of specific barcodes in lung metastases compared to the primary tumor (Fig. 3C). The intravenous injection of B16F10 cells resulted in lung metastases (Fig. 3D). A diverse composition of barcodes was observed in each lung metastasis, and the number of barcodes was less than that of barcodes in the original cells (*p* < 0.00076, Fig. 3E). We did not find barcodes enriched in metastases across all mice (Fig. 3F). The cellular composition of lung metastases was highly dependent on the mouse. Metastases in mouse #1 were composed of a few or multiple different cells, whereas those in mouse #4 and #5 comprised ~ 2000 different clones. Mice #2 and #3 showed metastases composed of a few diverse cell types (Fig. 3G). These data indicated the method-dependent cellular composition of B16F10 lung metastases. These results suggested that most B16F10 cells can contribute to metastasis formation.

**Fig. 4:**
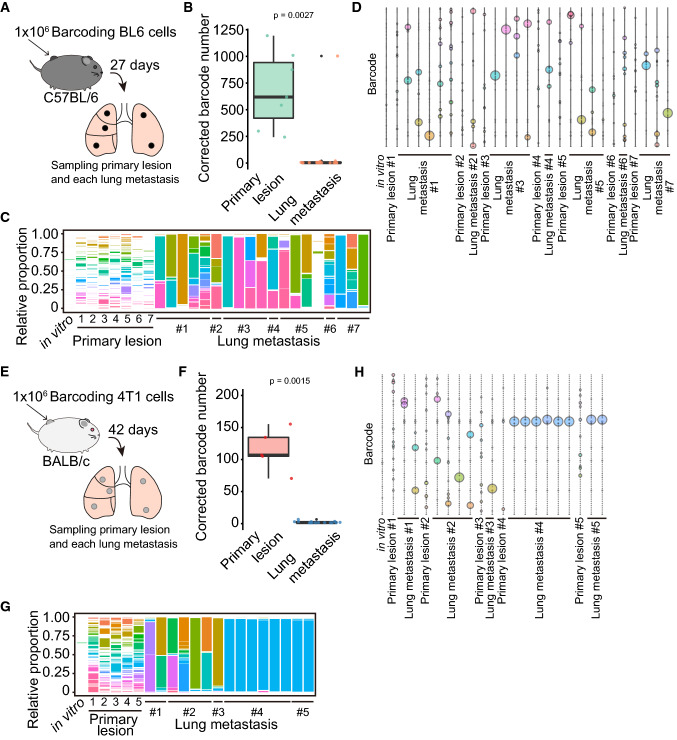
The formation of spontaneous lung metastases by a limited number of cell types. **A** Schematic diagram illustrating the establishment of C57BL/6N mice with the BL6 spontaneous lung metastases. Barcoded BL6 cells were inoculated on the back of C57BL/6N mice. The primary tumor and lung metastases were separately collected on day 27 after the inoculation. **B** Box plot of the corrected barcode number by Shannon entropy in BL6 primary tumor and lung metastases. *p* value was calculated by Welch’s *t*-test. **C** 100% stacked bar plot of the proportions of barcodes in the BL6-burdened mice. **D** The bubble plot of top 10 barcodes corrected from each of the 27 samples (*n* = 1 for in vitro, *n* = 7 for primary lesion, and *n* = 19 for lung metastasis) in the BL6-burdened mice. The total bubble number is 166 in each line since there is a redundancy. Bubble size indicates the proportion of the barcode. **E** Schematic diagram depicting the establishment of BALB/c mice with the 4T1 spontaneous lung metastases. Barcoded 4T1 cells were inoculated on the back of BALB/c mice. The primary tumor and lung metastases were separately collected on day 42 after the inoculation. **F** Box plot representing the corrected barcode number by Shannon entropy in 4T1 primary tumor and lung metastases. *p* value was calculated by Welch’s *t*-test. **G** 100% stacked bar plot of the proportions of barcodes in 4T1-burdened mice. **H** The bubble plot of top 10 barcodes corrected from each of the 21 samples (*n* = 1 for in vitro, *n* = 5 for primary lesion, and *n* = 15 for lung metastasis) in 4T1-burdened mice. The total bubble number is 75 in each line since there is a redundancy. Bubble size indicates the proportion of the barcode

To examine the cellular composition of spontaneous lung metastases, we barcoded BL6 and 4T1 cells since they can spontaneously establish lung metastases from the primary tumor. The barcoded BL6 cells contained ~ 3200 types of barcodes. We inoculated 1 ×  10^6^ barcoded BL6 cells onto the backs of C57BL/6N mice (Fig. 4A). The BL6 primary tumors comprised 683 barcodes on average (median, 747); in contrast, the lung metastases comprised 58 barcodes on average (median, 4) (*p* = 0.0027, Fig. 4B). Most lung metastases were composed of a few clones; however, some metastases contained ~ 1000 clones, suggesting that it caused a bias when the whole lung was analyzed to determine the cellular composition (Fig. 4C). Each metastasis was enriched in different clones in the same mice and across all the BL6-burdened mice (Fig. 4D). The primary tumors contained 114 barcodes on average (107 on median) although we inoculated the same number of barcoded-4T1 cells into BL6 cells (Fig. 4E and 4F). Most of the 4T1 spontaneous lung metastases contained two barcodes on average (median, two), which was significantly less than that in the primary tumor (*p* = 0.0015, Fig. 4F). All 4T1 lung metastases analyzed contained one or two clones (Fig. 4G). Mice #1 and #2 formed lung metastases from the different clones, whereas mouse #4 developed five metastases from the same clone (Fig. 4H). Moreover, one lung metastasis in mouse #4 and two metastases in mouse #5 were composed of the same clone, suggesting that 4T1 cells contain pre-existing cells with high metastatic ability. These data suggest that most spontaneous lung metastases are composed of a limited number of stochastically selected cells and that some cell lines, such as 4T1, have a rare population, which is consistent with a previous report [40].

**Fig. 5:**
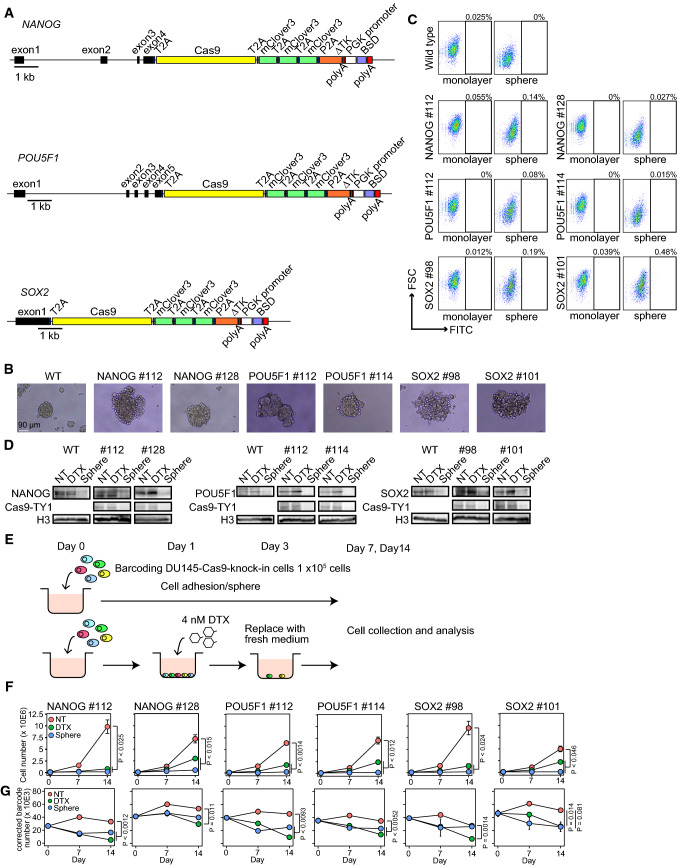
Establishing DU145 cells with stem cell-related transcription factors expression-recording system. **A** Schematic diagram illustrating Cas9 cassette insertion immediately before the stop codon of *NANOG*, *POU5F1*, and *SOX2*. The Cas9 sequence was ligated to the gene with the T2A peptide sequence. The Cas9 cassette included Cas9, three repeated mClover3, delta thymidine kinase (∆TK), and PGK promoter-driven blasticidin S-resistant gene (BSD). Cas9 and mClover3 were separated at the 2A peptide sequence after translation. **B** The representative images of wild-type and DNA-barcoded Cas9-introduced DU145 cells. **C** The dot plot of mClover3 expression analyzed by flow cytometer. **D** The western blots of NANOG, POU5F1 (OCT3/4), SOX2, and Cas9-TY1 (H3, loading control). Proteins obtained from 1 or 2 × 10^5^ cells were loaded in each lane (NT, non-treatment; DTX, docetaxel treatment; Sphere, sphere formation). **E** Schematic diagram illustrating the collection of DTX-resistant DU145 cells. **F** The line plot of cell proliferation of DNA-barcoded Cas9-introduced DU145 cells (*n* = 3). **G** The line plot of the corrected barcode number in DNA-barcoded Cas9-introduced DU145 cells on days 0, 7, and 14 (*n* = 3)

The DNA barcoding system can identify diverse cell proliferation, anticancer drug-resistant cells, and cell metastasis. However, it cannot trace the lineage of cells with specific gene expression. CSCs are thought to contribute to cancer progression. However, the descendants of CSCs comprise a significant population of cancer cells after anticancer drug treatment and under cell stress conditions is not clear. Hence, we used Cas9 and stgRNA to establish transcription-recording cancer cells [27]. We previously found that DU145 cells (human prostate cancer cell line) require the expression of NANOG, a stem cell-related transcription factor, to resist anticancer drugs and escape immune surveillance [13, 31]. We inserted the Cas9 sequence along with three repeats of mClover3 immediately before the stop codon of stem cell-related transcription factors, including *NANOG*, *POU5F1*, and *SOX2* (NANOG-, POU5F1-, and SOX2-DU145 cells) (Fig. 5A). DU145 cells with the Cas9 cassette in each gene formed spheres similar to wild-type cells (Fig. 5B). Although we did not detect significant mClover3-positive cells in NANOG-DU145, POU5F1-DU145, and SOX2-DU145 cells by flow cytometer (Fig. 5C), we detected Cas9 protein in control or Docetaxel (DTX)-treated NANOG-DU145, POU5F1-DU145, and SOX2-DU145 cells by western blotting (Fig. 5D). DU145 cells with the Cas9 cassette in each gene expressed transcription factors comparable to those in wild-type cells (Fig. 5D). DTX- treatment did not significantly affect the protein expression of each transcription factor but sphere formation decreased the protein expression (Fig. 5D and E). These results indicate that the Cas9 cassette acts as a surrogate for the expression of each transcription factor in DU145 cells with Cas9 cassette.

**Fig. 6:**
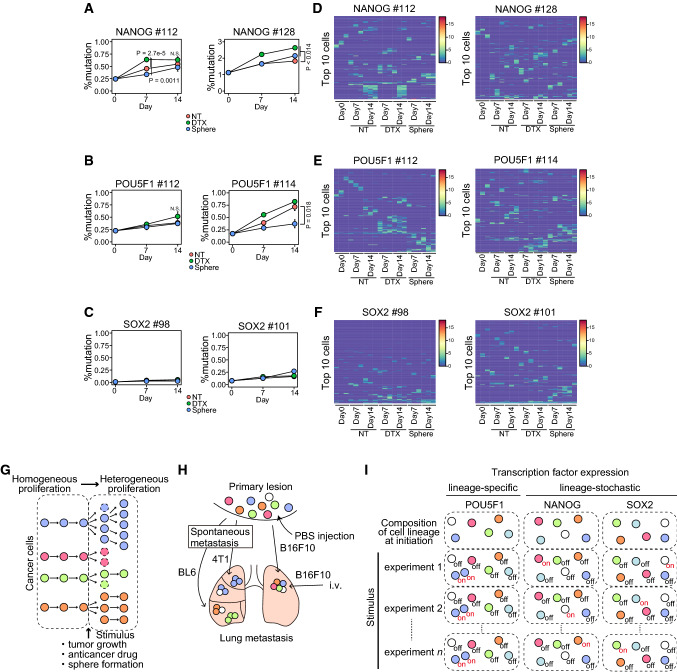
Tracing a progeny of cells expressing stem cell transcription factors, including NANOG, POU5F1, and SOX2, in anticancer drug treatment or sphere formation. **A**–**C** Line plot depicting the percent mutation in stgRNA on days 0, 7, and 14 in NANOG-DU145 (**A**), POU5F1-DU145 (**B**), and SOX2-DU145 (**C**) cells. *P* value was calculated using Welch’s *t*-test with Bonferroni adjustment (*n* = 3). **D**–**F** Heatmap representing the average score in each cell of top 10 cells with a high number of mutated reads from each sample in NANOG-DU145 (*n* = 196 for #112; *n* = 176 for #128) (**D**), POU5F1-DU145 (*n* = 195 for #112; *n* = 203 for # 114) (**E**), and SOX2-DU145 (*n* = 152 for #98; *n* = 198 for #101) (**F**) cells. **G** Schema of the composition of cancer cells during tumor progression. **H** Schema of method- and cell-type-dependent variations in lung metastasis formation. **I** Schema of the lineage-specific or -stochastic expression of transcription factors. *NT* non-treatment, *DTX* docetaxel treatment; and *Sphere* sphere formation

To determine whether the expression of these transcription factors promotes resistance to anticancer drugs and stress conditions, we traced the expression of transcription factors in each cell during DTX treatment or sphere formation. We labeled the Cas9 cassette-introduced DU145 cells with a lentivirus-encoding DNA barcode and stgRNA. We identified 116,722 barcodes corrected using Shannon entropy and Hamming distance 2. DTX treatment and sphere formation suppressed cell proliferation in all DU145 cells with the Cas9 cassette and DNA barcoding (Fig. 5F). The number of DNA barcodes in each cell line was decreased on DTX treatment compared to that in untreated cells (Fig. 5G). Sphere formation reduced the number of DNA barcodes in NANOG #112 and POU5F1 cells but not in NANOG #128 and SOX2 cells (Fig. 5G). Next, we analyzed the sequence changes that occurred in stgRNA. We confirmed a mutation at position 16 in the stgRNA sequence in DU145 cells with Cas9 compared to the plasmid control (Fig. S1), indicating that DU145 cells with Cas9 and stgRNA work to record transcription factor expression levels in the stgRNA. We used deletion and insertion mutations for the calculation because mismatch mutations were found throughout the stgRNA sequence, suggesting a Cas9-independent error. Thus, we compared deletion and insertion as percent mutations in stgRNA on days 0, 7, and 14 in each cell. The percentage of mutated stgRNA increased in DTX-treated NANOG-DU145 cells (Fig. 6A). In contrast, we detected a slight increase in mutated stgRNA in DTX-treated POU5F1-DU145 cells (Fig. 6B) but not in SOX2-DU145 cells (Fig. 6C). However, sphere formation did not significantly increase the percentage of stgRNA mutations in any of the cells (Fig. 6A–C).

To examine whether the expression of these transcription factors is associated with an increase in the proportion of cells, we computed Spearman's correlations between the mean of mutation size of stgRNA and the number of barcode reads, weighted by the number of barcode reads. We then detected higher and significant values of the correlations, especially in Day14 DTX-treated POU5F1 cells (Spearman’s correlation 0.455 for #112 and 0.433 for #114), compared to NANOG (Spearman’s correlation 0.410 for #112 and 0.388 for #128) and SOX2 (Spearman’s correlation 0.350 for #98 and 0.370 for #101) cells (Fig. 6D–F, Table S1). The results suggest that cells with POU5F1 expression are associated with an increase in proportion compared to NANOG and SOX2 expression. Next, we examined whether a cell with a specific lineage expresses these transcription factors, particularly in the top 10 cells with a high number of mutated reads. POU5F1-DU145 cells included more cells that are susceptible to recurrently acquiring the mutation during DTX treatment or sphere formation (Fig. 6E). Compared to the POU5F1 expression profile, NANOG and SOX2 tended to be more stochastically expressed in DU145 cells (Fig. 6D and F). The cumulative distribution fraction of the mutation in all cells showed no significant difference between samples in each cell, as mutations in stgRNA were not often detected (Fig. S2). We also did not detect significant changes in the cellular composition during DTX treatment or sphere formation (Fig. S3). These results suggest that POU5F1 is expressed in a specific cell population upon distinct stimulation in DU145 cells.

## Discussion

### Anticancer therapy effect on tumor composition

Tumors are composed of various types of cancer cells. Whether the composition of cancer cells is consistent or increases in heterogeneity during tumor growth remains unclear. Our DNA-barcoded B16F10 cells showed a homogeneous proliferation within 10 days after inoculation of cancer cells and subsequently switched to a heterogeneous proliferation state 14 days after inoculation and decreased the number of cell variations (Fig. 6G). These results suggest three possibilities: (1) Rapid tumor growth induces hypoxia, resulting in stochastic cell death at a later stage of tumor growth; (2) cell–cell competition includes stochastic cell death in a loser cell since there is limited space in the mouse skin; and (3) Immune cells begin to eliminate cancer cells at a later stage of tumor growth.

Although DTX and HVJ-E treatments significantly suppressed tumor growth, neither decreased the barcode number in any tumor (Fig. 2). The remaining cancer cell profile remains controversial; several studies suggest that chemotherapy eradicates most cancer clones [2, 14, 16, 19, 33] or ~ 80% of clones survive after chemotherapy in the PDX model [24]. This discrepancy may be caused by a difference in sensitivity to detect barcodes or a difference in cancer cells, although further experiments are required. We calculated the JS divergence score using the top 10 barcodes from each sample or all barcodes to evaluate the effect of low-frequency barcodes. The top 10 barcodes showed that DTX treatment increased heterogeneity more than HVJ-E treatment; however, the difference became weak when we used all barcodes. Low-frequency barcodes may become noise to produce this difference. Another possibility is that, since HVJ-E was administered intratumorally, it is expected to act uniformly on tumor cells. However, since DTX is transported into the tumor via intratumor blood vessels, its concentration may be differing between the vicinity of blood vessels and distal to blood vessels, resulting in more significant cellular heterogeneity. Our findings provide critical information for screening experiments using a gRNA library to analyze the proportion of cancer cell clones in vivo.

### Method- and cell-type-dependent variations in lung metastasis formation

Whether a specific population in a primary tumor has metastatic ability or whether cancer cells acquire this ability stochastically remains unclear. We found that different methods of generating lung metastasis models, including intratumor injection of PBS, intravenous injection, and spontaneous lung metastasis models, have a significant impact on the composition of cells that form metastasis (Fig. 6H). The number of barcodes in lung metastasis developed by intra-tumoral injection of PBS was comparable to that of the primary tumor, suggesting that the injection causes physical damage and forms a relatively large tumor mass that enters the blood vessel and generates a metastatic lung lesion. The intravenous injection of B16F10 cells generates various compositions of lung metastases in an individual-dependent manner, probably because the probability that the migrating cancer cells are reached each lesion is dependent on the histological location, e.g., distance from a blood vessel, resulting in lung metastases with the different clonal variations.

Spontaneous lung metastasis models using BL6 and 4T1 cells generated lung metastases comprising four or two cells, respectively (Fig. 4). Although most lung metastases of BL6 cells were formed by independent cells, 4T1 cells may have cells that are highly capable of developing lung metastases. Reportedly, 27% of melanoma cells can metastasize, and some 4T1 cells are prone to invade into blood vessels [30, 40]. Taken together, our findings suggest that 4T1 cells have the ability to form cell populations with different properties and comprise elite cells with metastatic capacity.

### CSC properties in DU145 human prostate cancer cell line

WE traced the progeny of cells expressing stem cell transcription factors, including NANOG, POU5F1, and SOX2, during anticancer drug treatment or sphere formation. We observed that cells retrospectively expressing NANOG were increased by DTX treatment at some time point, but those retrospectively expressing POU5F1 and SOX2 were not (Fig. 6I). However, DTX treatment or sphere formation induced the expression of POU5F1 but not NANOG and SOX2 in identical cells. Notably, distinct cells showed increased POU5F1 expression in response to DTX treatment or sphere formation, suggesting that POU5F1 is expressed in a scarce population and that the population includes various cells that respond differently to external stimuli. POU5F1 expression is controlled by several transcription factors, such as HIF1 and MYC, in cancer cells. In renal cell carcinoma (RCC), POU5F1 expression is activated by a HIF1-pathway-responsive promoter in the long terminal repeat (LTR) element that is located upstream of POU5F1 [34]. DNA methylation at the MYC-binding elements in the POU5F1 locus is involved in regulating POU5F1 expression in cancer cells [5]. These suggest that epigenetic status at the POU5F1-regulating element region is critical for the regulation of POU5F1 expression. DTX- or sphere-responding POU5F1-DU145 cells might have a distinct epigenetic profile in the POU5F1-regulating element region. However, DTX- or sphere-responding POU5F1-DU145 clones are 0.52% and 0.37% for #112 and 0.82% and 0.37% for #114 in DU145 cells, meaning it is challenging to analyze a property of the rare population even using current single-cell technologies. NANOG expression is regulated by several transcription factors, such as p53, STAT3, Gli1/2, and HIF [8]. SOX2 expression is regulated by SOX4, OCT4, STAT3, E2F, DNMT, and FoxO1 [41]. Thus, the expression of these cancer stem-like cell-related transcription factors is regulated by their own distinct systems. These imply that cancer cells have multiple way to generate cancer stem-like cells to survive under various stresses, such as anticancer drugs. These findings suggest the following: (1) A rare population of DU145 cells express stem cell transcription factor; and (2) POU5F1 is reproducibly expressed in specific cells depending on the type of stimulus.

Consistent with the gene expression tracing data showing transcription factor expression in a rare proportion of cells, the protein expression levels of NANOG, POU5F1, and SOX2 per cell were not increased by DTX treatment and sphere formation. These results contradict previous findings that sphere formation increases stem cell transcription factor expression [11]. The difference may be due to the presence of pseudogenes that are expressed in cancer cells [11, 13] or a decrease in the total amount of protein in sphere-formed cancer cells.

We could only analyze the retrospective stem cell transcription factor expression in vitro because our Cas9-introducing DU145 cells could not form tumors even in immunodeficient NSG mice. Thus, it is unclear whether our findings are consistent with in vivo scenarios. Although further experiments are required to demonstrate the roles of stem cell transcription factors in tumor progression, our results suggest that POU5F1 has unique features, at least in the DU145 cell line.

### Study limitations

Our findings are based on barcoding cancer cells; thus, it is unclear whether we can expect to apply cancer clonal behavior to actual cancer patients. The mechanisms that generate different clonal behaviors in cancer cell lines are still unknown. Mouse embryonic stem cells exhibit high or low Nanog expression, which is accounted for by a network of Pou5f1 and Sox2 [12]. Compared to embryonic stem cells, cancer cells express deficient expression of transcription factors in a small proportion of cells. It is unclear whether cancer cells utilize the exact mechanisms to regulate NANOG expression. Further experiments are required to elucidate mechanisms that maintain the same proportion of cancer cells with unique properties.

## Conclusion

By combining clone tracing with retrospective gene expression analysis, we found that some cancer cell lines contain a small proportion of cells with high metastatic ability and unique properties in response to anticancer drug treatment and sphere formation. In future, the development of new technologies to analyze the history of transcription and epigenome without knock-in of Cas9 will allow us to perform high-throughput analysis of retrospective gene expression and identify essential genes for tumor progression and potential therapeutic targets.

## Methods

### Cell culture

B16F10 murine melanoma, 4T1 mammary carcinoma, and DU145 (androgen-independent human prostate) cell lines were purchased from the American Type Culture Collection (Rockville, MD, USA). B16BL6 murine melanoma cell line was a gift from Dr. Yoshio Okada. Human embryonic kidney (293FT) cells were purchased from Thermo Fisher Scientific. B16F10 and DU145 cells were maintained in Dulbecco’s Modified Eagle’s Medium (DMEM) (Nacalai Tesque Inc., Tokyo, Japan) supplemented with 10% fetal bovine serum (FBS) (Biowest, Nuaillé, France), 100 U/mL penicillin and 0.1 mg/mL streptomycin (Penicillin–Streptomycin Mixed solution) (Nacalai Tesque). 4T1 and B16BL6 cells were maintained in RPMI-1640 medium (Nacalai Tesque) supplemented with 10% FBS, 100 U/mL penicillin, and 0.1 mg/mL streptomycin. 293FT cells were maintained in DMEM supplemented with 10% FBS, 1 mM sodium pyruvate (Nacalai Tesque), 2 mM l-glutamine (Nacalai Tesque), and 1% nonessential amino acids (Nacalai Tesque). G418 (500 μg/mL) was added to a subculture of 293FT cells. The cells were incubated at 37 °C in a humidified atmosphere containing 5% CO_2_.

### Plasmid construction

pX330 (Addgene) was treated with BbsI (New England Biolabs), and DNA oligonucleotides comprising a gRNA sequence targeting the 3′ untranslated region (NANOG and SOX2) or downstream of the gene (POU5F1) were inserted into the BbsI site (NANOG: CCCATCCCTCATAGGATTTT, SOX2: GTACTGGCGAACCATCTCTG, and POU5F1: TTAAGGTCACACAACATCAG).

The 5′ and 3′ homology arms of NANOG, SOX2, and POU5F1 were amplified from the genomic DNA of DU145 cells by PCR using KOD FX Neo (TOYOBO). The 5′ and 3′ homology arms were subsequently linked together using PCR. MfeI and AscI sites were inserted between the 5′ and 3′ homology arms using PCR. This fragment was then subcloned into the Zero Blunt TOPO vector (Thermo Fisher Scientific). This plasmid was digested with PmeI (New England BioLabs), and DNA fragments containing homology arms were ligated into the pMA vector (pMA-NANOG/SOX2/POU5F1-armV2-MfeIAscI). A cassette containing Cas9 was inserted between the MfeI and AscI sites of pMA-NANOG/ SOX2/ POU5F1-armV2-MfeIAscI.

For the construction of the stgRNA expression vector (pKLV2-20nt2-stgRNA), the fragments containing stgRNA (20nt-2) and the stgRNA scaffold were cloned into the BbsI and *Bam*HI sites of pKLV-U6gRNA(BbsI)-PGKpuro2ABFP.

### Barcode library construction

A 77-mer oligonucleotide containing the barcode sequence was purchased from Thermo Fisher Scientific or Integrated DNA Technology. The barcode structure used was a 30-base sequence, comprising 10 repeats of W (weak base: A or T) -S (strong base: G or C) -N (A, T, G, or C). PCR was performed using this 77- mer single-stranded oligonucleotide and two types of primers with the Q5 Hot Start High-Fidelity 2 × Master Mix (New England BioLabs) to generate double-stranded oligonucleotides. PCR products were digested with EcoRI (New England BioLabs) and BamHI (New England BioLabs) in the presence of rSAP (New England BioLabs) and ligated into the EcoRI and BamHI sites of pKLV2-20nt2-stgRNA using Ligation high Ver.2 (TOYOBO). The ligated products were transformed into Stbl4 competent Escherichia coli cells using the Gene Pulser Xcell Electroporation System (Bio-Rad) and cultured overnight in LB medium containing 50 μg/mL carbenicillin. Plasmid DNA was extracted using the Zymopure II Plasmid Maxiprep Kit (Zymo Research).

### Lentivirus production

293FT cells were seeded into a 10 cm culture dish at a density of 5 × 10^6^ cells/dish and incubated for 24 h. Before transfection, the 293FT cells were replaced with fresh medium (5 mL/dish). A: Lentiviral vector (5.4 μg/dish), psPax2 (5.4 μg/dish, Addgene), and pMD2.G (1.2 μg/dish, Addgene) were mixed in Opti-MEM (1.5 mL/dish, Thermo Fisher Scientific). B: PEI Max (50 μL/dish) was added to Opti-MEM (1.5 mL/dish). Samples A and B were incubated for 5 min at room temperature. A and B were then mixed and incubated for 20 min at room temperature (DNA/PEI max complex). After incubation, the DNA/PEI max complex was added to 293FT cells (3 mL/dish) and replaced with fresh medium (10 mL/dish) 18 h after transfection. The viral supernatant was harvested 48 h after transfection, filtered through a 0.45 μm membrane filter (Millipore), and stored at − 80 °C.

### Generation of DU145-Cas9 knock-in cells

Before transfection, plasmid DNA encoding Cas9 and antibiotic resistance genes (blasticidin or neomycin) was digested with PmeI (New England BioLabs). Each digested plasmid DNA (1 µg fragment each containing blasticidin and neomycin resistance genes) and 1 µg pX330 plasmid coding gRNA targeting the 3′ untranslated region or downstream of *NANOG*, *SOX2*, or *POU5F1* were transfected into the suspended DU145 (2.6 × 10^5^ cells/40 μL) using the Neon Transfection System (Invitrogen) (voltage: 1000, width: 30; pulse number: 2), and cultured in a 10 cm dish. After 2 days of transfection, blasticidin (7.5 μg/mL) and G418 (150 μg/mL) were added to a 10 cm dish. Single colonies were selected and individually cultured in 96-well plates followed by 24-well plates. Half of the sub-confluent cells were cryopreserved in CELLBANKER 1 (Takara Bio). The remaining cells were used to extract genomic DNA by heating at 95 °C for 10 min in the presence of 50 mM NaOH followed by the addition of 0.1% volume of 1 M Tris–HCl (pH 8.0) for neutralization. Genotyping PCR was performed using KOD FX Neo (TOYOBO).

### Cell barcoding

B16F10 (2 × 10^4^ cells/mL), 4T1 (5 × 10^4^ cells/mL), B16BL6 (5 × 10^4^ cells/mL), and DU145-Cas9 knock-in cells (1 × 10^5^ cells/mL) were seeded into 10 or 15 cm culture dishes and incubated for 24 h. Cells were then infected with the barcode library at an multiplicity of infection (MOI) of < 0.3 to be labeled with a single barcode per cell. The medium was replaced with fresh medium 24 h after infection, and the cells were incubated further for 24 h. MOI was assessed by the proportion of blue fluorescent protein (BFP)-expressing cells using flow cytometry (BD FACS Canto II). The cells were maintained in the presence of puromycin for 7 days to remove uninfected cells 48 h after infection.

### Sphere-forming culture

The spheres of barcoded-DU145-Cas9 knock-in cells were formed as previously described [31]. Briefly, each cell line (1 × 10^5^ cells) was plated on EZ-Bind Shut™ SP 100 mm Dish (IWAKI) or Prime Surface 60 mm dish (Sumitomo Bakelite) and cultured in DMEM/F12 (Gibco, NY, USA) supplemented with B27 (Gibco), 4 μg/mL insulin (Sigma, MO, USA), 20 ng/mL epithelial growth factor (Gibco), and 20 ng/mL basic fibroblast growth factor (ORF, Kopavogur, Iceland) for 7, 10, or 14 days at 37 °C and 5% CO_2_.

### In vitro treatment with DTX

DU145 Cas9 knock-in cells were seeded into 6-well plates at a density of 1 × 10^5^ cells/well on day 0 and incubated for 24 h. Media were subsequently replaced with fresh media containing 4 nM DTX (Tokyo Chemical Industry) and incubated for 48 h. Cells were retained in the absence of DTX after 48 h of incubation and collected on days 7 and 14.

### Mice

Six-week-old female C57BL/6N and BALB/c mice were purchased from CLEA Japan and maintained in a temperature-controlled pathogen-free room. All animals were handled according to the approved protocols and guidelines of the Animal Committee of Osaka University (Osaka, Japan).

### Preparation of HVJ-E

HVJ (VR-105 parainfluenza Sendai/52 Z strain) was acquired from the American Type Culture Collection (Manassas, VA, USA), amplified by injection into 10–14-day-old embryonated eggs and purified as previously described [26]. The purified live HVJ virus was inactivated by UV irradiation (189 mJ/cm^2^). The inactivated HVJ was used as HVJ-E.

### Tumors with different size

C57BL/6N mice were inoculated intradermally with barcoded-B16F10 cells (5 × 10^5^ cells/mouse). When the tumor diameter reached approximately 5, 10, or 15 mm, the mice were sacrificed and tumors were collected. Cell suspensions from tumors were obtained by mincing and digestion with collagenase type I (FUJIFILM Wako Pure Chemical Corporation), as previously described [26]. Viable cells were counted using trypan blue (Nacalai Tesque). Tumor size was measured using a slide caliper, and tumor volume was calculated using the following formula: tumor volume (mm^3^ = 0.5 × length (mm × [width (mm)]^2^)).

### Tumor treatment

C57BL/6N mice were inoculated intradermally with barcoded-B16F10 cells (5 × 10^5^ cells/mouse). When the tumor diameter reached 4.3–4.8 mm, PBS (Nacalai Tesque), HVJ, or DTIC (FUJIFILM Wako Pure Chemical Corporation) was administered. PBS (50 μL/shot) and HVJ (2000 HAU/50μL/shot) were injected directly into the tumor thrice every alternate day. DTIC (100 mg/kg) was administered intraperitoneally thrice every alternate day. Ten days after the first administration, the mice were sacrificed and tumors were collected. Cell suspensions from the tumors were obtained by mincing and digestion with collagenase type I, as previously described [26]. The number of viable cells was counted using trypan blue.

### Intravenous lung metastasis model

Barcoded-B16F10 cells (5 × 10^5^ cells) were suspended in 200 μL of PBS (Nacalai Tesque) and injected into the tail vein of C57BL/6N mice. Eight days after the injection of cells, the mice were sacrificed, and macroscopically visible lung metastases were collected.

### Spontaneous lung metastasis model

To establish the 4T1 lung metastasis model, barcoded-4T1 cells (1 × 10^6^ cells) were implanted subcutaneously into BALB/c mice. The primary tumors and macroscopically visible lung metastases were collected 42 days after tumor inoculation.

To produce the B16BL6 lung metastasis model, barcoded-B16BL6 cells (1 × 10^6^ cells) were implanted subcutaneously in C57BL/6N mice. The primary tumors and macroscopically visible lung metastases were collected 27 days after tumor inoculation.

Cell suspensions from primary tumors were obtained by mincing and digestion with collagenase type I as previously described [26]. The number of viable cells was counted using trypan blue.

### Next-generation sequencing of barcode library

Genomic DNA was isolated using the DNeasy Blood & Tissue Kit (Qiagen) or the Quick-DNA™ Miniprep Plus Kit (Zymo Research). The region containing the barcode and stgRNA was amplified using Q5 Hot Start High-Fidelity 2 × Master Mix (New England BioLabs). For the PCR of primary tumor of Barcode-B16F10 cells, genomic DNA isolated from 2 × 10^6^ cells was used. When the number of harvested barcoded-B16F10 cells was less than 2 × 10^6^ cells, all harvested cells were used. All genomic DNA isolated from each lung metastasis was used for the PCR of lung metastasis. For the PCR of the primary tumors of barcoded-4T1 and barcoded-B16BL6 cells, genomic DNA isolated from 4 × 10^6^ cells was used. For the PCR of DU145 cells, half of the harvested genomic DNA was used. The following cycling conditions were applied for PCR: 30 s at 98 °C; 30 cycles of 10 s at 98 °C, 15 s at 61 °C, and 20 s at 72 °C; and a final extension at 72 °C for 2 min. The following primer pairs were used for the first PCR:

gLibrary-Hiseq-50 bp-SE-U1: ACACTCTTTCCCTACACGACGCTCTTCCGATCTTGTGGAAAGGACGAAACA

gLibrary-HiseqX-L1: GACTGGAGTTCAGACGTGTGCTCTTCCGATCTCTAAAGCGCATGCTCCAGAC

The PCR products were purified using Monarch PCR & DNA Cleanup Kit (New England BioLabs) or NucleoSpin Gel and PCR Clean-up Midi (MACHEREY–NAGEL GmbH & Co., KG).

Eight hundred picograms of purified first PCR products was used as templates for the second PCR with NEB Next Q5 Hot Start HiFi PCR Master Mix (New England BioLabs) in a total volume of 50 μL. In the second PCR, each sample was labeled with 6-bp-long index sequences. The PCR cycling conditions were as follows: 30 s at 98 °C; 12 cycles of 10 s at 98 °C and 1 min 15 s at 65 °C; and a final extension for 5 min at 65 °C. The following primer pairs were used for the second PCR:

CRISPR-Library-F: AATGATACGGCGACCACCGAGATCTACACTCTTTCCCTACACGACGCTCTTCCGATOT

iPCRtag-X-L2: CAAGCAGAAGACGGCATACGAGATNNNNNNGTGACTGGAGTTCAGACGTGTGCTCTTCCGATOT

PCR products were purified using AMPure XP (Beckman Coulter). DNA concentrations of the PCR products were assessed by qPCR using the KOD SYBR qPCR Mix (TOYOBO). Subsequently, PCR products were pooled at equimolar ratios, and sequencing was performed with 30% PhiX on an Illumina HiseqX with 150 bp paired-end reads.

### Western blot analysis

Cell lysates were subjected to SDS-PAGE, and the separated proteins were transferred onto Immobilon-P transfer membranes (Merck Millipore, Tokyo, Japan). To detect the proteins, anti-NANOG [Cell Signaling Technology (CST), MA, USA; #4903], anti-OCT4 (CST; #2750), anti-SOX2 (CST; #3579), anti-Ty1 (Sigma-Aldrich, SAB4800032), and anti-histone H3 (CST; 4499) primary antibodies were used. Enhanced chemiluminescence horseradish peroxidase-conjugated donkey antirabbit IgG (GE Healthcare Japan, Tokyo, Japan) was used as the secondary antibody for the detection of NANOG, POU5F1, SOX2, and histone H3. Signals were detected using Chemi-Lumi One (Nacalai Tesque) or Chemi-Lumi One Super (Nacalai Tesque) and an ImageQuant LAS 4000 mini system (GE Healthcare).

### Bioinformatic analysis of data

#### Tools

Awk v4.1.3 (https://www.gnu.org)

Cutadapt v1.9.1 [22]

FASTX-Toolkit 0.0.13 (http://hannonlab.cshl.edu/fastx_toolkit/)

trim_galore v0.6.6 (https://www.bioinformatics.babraham.ac.uk/projects/trim_galore/)

bbmap v38.90 (https://www.osti.gov/servlets/purl/1241166)

umi_tools v1.0.1 [35]

R 4.0.4 (https://www.r-project.org)

Rstudio 1.4.1106 (https://www.rstudio.com)

WeightedCorr v2.1 (https://github.com/matthijsz/weightedcorr)

#### R package

Dplyr v1.0.5 (https://cran.r-project.org/web/packages/dplyr/index.html)

ggplot2 v3.3.3 (https://cran.r-project.org/web/packages/ggplot2/index.html)

tidyverse v1.3.0 (https://cran.r-project.org/web/packages/tidyverse/index.html)

gplots v3.1.1 (https://cran.r-project.org/web/packages/gplots/index.html)

#### NGS data of barcodes in B16F10, B16BL6, and 4T1 tumors

The adapter sequence was removed from the read2 sequence using cutadapt with the option -e 0.2. Reads with low quality and < 25 bases were removed using fastq_quality_trimmer with the options -Q33 -t 20 -l 25 and -Q33 -q 20 -p 80. Fastq was converted to Fasta using fastq_to_fasta with the option -Q 33. Next, we counted the reads of each barcode variety in each sample, accepting only length-30 barcode sequences. Although not all of them had the exact form of WSN (W: A/T, S: C/G, N: A/T/G/C) × 10, we did not impose restrictions on that point (in fact, exact WSNx10 reads account for only 10% of the total, and limiting to them would destroy essential parts of our data). The error might be caused by the limitation of a company's ability to generate the precise DNA oligo. Reads with unidentified bases were excluded from the analyses. Since each sample included many similar barcodes, due to reading errors or mutations, we merged them if the Hamming distance was ≤ 2. The algorithm is outlined in (a). The resulting dataset was used in the subsequent analyses. This reduced the variation to approximately 10–50%, depending on the origin of the samples.
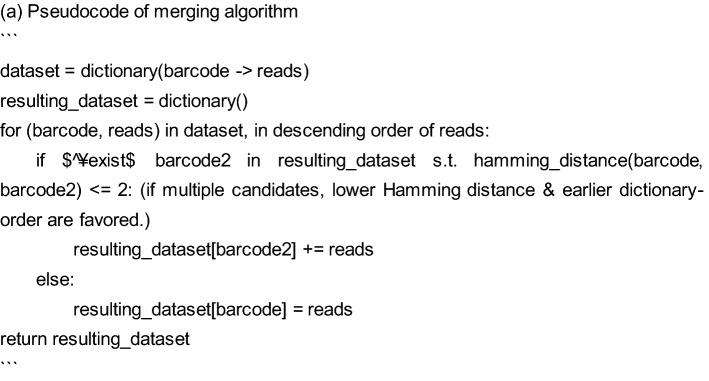


#### DU145 Expression-Records

1. Expression scores
  Low-quality reads were removed from the paired reads using trim_galore with the option –fastqc -j 6 –paired. Paired reads were combined using bbmerge. stgRNA and barcode sequences were extracted using umi_tools with the option –bc-pattern to determine a user-defined barcode pattern. To estimate the cumulative expression levels of the target gene, we adopted mutation scores from the original stg sequences [27]. We scored a mismatch base as 1, a start of deletion or insertion as 2.5, and an extension of them as 0.5. Alignment with the minimum mutation score was computed using the conventional dynamic planning algorithm. For even-score alignments, mutations were preferred in the following order: mismatch, insertion, and deletion. The obtained alignments gave separate scores for mismatches, insertions, and deletions. Among them, the mismatch scores presented rather noisy behaviors in our data; therefore, we employed only insertion and deletion scores as indicators of the expression levels.
2. Valid stg selection
  The stg sequences should ideally be unique in the barcoded-plasmids inserted into DU145 cells. However, the real sequence data reported additional 1386 stg variations, accounting for 5.2% reads in addition to the correct template (94.8% reads). Some of these variations were very different from the template and produced large noise in the stg sequences of the resulting cells. To delete them, we picked up variations with 4.0 or larger mutation scores from the template, including 143 variations counting 215 reads (0.00068% of the total). For each of the 71,361 stg variations that were observed in any of the samples, we computed the mutation scores from each of the 143 variations, and if any of them was smaller than that from the template, the stg variation was regarded as originating from a wrong-stg plasmid and the corresponding reads were simply ignored. In this way, 46,134 were deleted and 25,227 stg variations survived. The ignored reads were 0.3–7.4% in each sample.
3. Barcode selection and merger
  Similar to the tumor data, including the stg sequences, a set of (barcode, stg, and reads) triplets were obtained for each sample. The barcodes in the original plasmids were selected and merged using the same algorithm as for the tumor data. For example, (A, X, M) and (A′, X, N) were merged into (A, X, M + N), whereas (A, X, M) and (A′, Y, N) yielded (A, X, M) and (A, Y, N), when A and A′ were similar barcodes (Hamming distance ≤ 2). For all other samples, we retained only reads with barcodes included in the resulting set. After this process, the barcode variations were reduced to approximately 25% (plasmid), 50% (total), 40% (Day 0), 20–30% (Day 7), and 10–25% (Day 14).

#### Data processing using R

Further analysis of barcode data was performed using R. To remove barcodes derived from sequence error, barcodes detected in all plasmids, barcoded-B16F10, BL6, and 4T1 cells were analyzed using R package dplyr. The corrected barcode number of each sample was calculated as previously reported and plotted using the R ggplot2 package [4].

#### Analysis of barcode composition

For the comparison of the barcode composition between samples, the JS divergence was calculated using all the barcodes or the top 10 barcodes of each sample, and a heatmap was generated using the R ggplot2 package. A bubble plot of the top 10 barcodes for each sample was constructed using the R ggplot2 package. A cumulative bar plot of the barcodes was constructed using the R ggplot2 package.

#### Analysis of stgRNA

Hierarchical clustering heatmaps of mean mutation scores were constructed using the top 10 barcodes with mutated reads using the R gplot package. A heatmap of the mutation rate (deletion, insertion, and mismatch) in each position in stgRNA was constructed using the R ggplot2 package. The cumulative distribution fractions of insertions and deletions in each sample were plotted using the R ggplot2 package.

### Statistical analysis

Statistical analysis was performed using R. Welch’s *t*-test with Bonferroni adjustment was used to compare the two samples. Weighted Spearman correlation was calculated WeightedCorr. Statistical significance was set at *p* < 0.05.

### Supplementary Information

Below is the link to the electronic supplementary material.

Supplementary file1 (PDF 28401 KB)

## Data Availability

The barcoding datasets generated during this study are available from the corresponding authors upon reasonable request. The codes used in this study are available at GitHub (https://github.com/GeneTherapyScience/barcode_expression/).

## Abbreviations

CSCs: Cancer stem-like cells
PDXs: Patient-derived xenografts
BL6: B16/BL6
stgRNA: Self-targeting guide RNA
JS: Jensen–Shannon
HVJ-E: Hemagglutinating virus of Japan envelope
DTIC: Dacarbazine
DTX: Docetaxel
RCC: Renal cell carcinoma
LTR: Long terminal repeat
CDF: Cumulative distribution fraction
∆TK: Delta thymidine kinase
BSD: Blasticidin S-resistant gene
NT: Non-treatment
