# Supplementary material for: DNA barcoding and gene expression recording reveal the presence of cancer cells with unique properties during tumor progression
Source: Cell Mol Life Sci. 2022 Dec 23;80(1):17. doi: 10.1007/s00018-022-04640-4 (PMC9789022; doi:10.1007/s00018-022-04640-4)
Supplement: Supplementary file 1 — Supplementary file1 (PDF 28401 KB) [file 18_2022_4640_MOESM1_ESM.pdf]

Supplementary Data

DNA barcoding and gene expression recording reveal the presence of cancer cells with unique properties during tumor progression

Yuka Umeki, Noriaki Ogawa, Yuko Uegaki, Kotaro Saga, Yasufumi Kaneda, and Keisuke Nimura

Supplementary Figure legends

Figure S1. Evaluation of mutation in stgRNA

Heatmap depicting mutation rate (deletion, insertion, and mismatch) in each position in stgRNA. NT, non-treatment; DTX, docetaxel treatment; and Sphere, sphere formation

Figure S2. The detection rate of mutation in stgRNA in all cells

The cumulative distribution fraction of insertion and deletion in each sample. NT, non-treatment; DTX, docetaxel treatment; and Sphere, sphere formation

Figure S3. The analysis of cellular composition in DU145 cells

(**A**) The bubble plot of top 10 barcodes corrected from each of the 21 samples (n = 3 for each sample). The total bubble number is 97 for NANOG #112, 83 for NANOG #128, 99 for POU5F1 #112, 92 for POU5F1 #114, 97 for SOX2 #98, and 94 for SOX2 #101 in each line since there is a redundancy. Bubble size indicates the proportion of the barcode. (**B**) Heatmap representing JS divergence calculated using top 10 barcodes in each sample or all barcodes. NT, non-treatment; DTX, docetaxel treatment; and Sphere, sphere formation

Figure S4. Raw images of Western blots

Table S1. The mean significant Spearman's correlations between mutation size of stgRNA and weighted number of cell barcode reads in DU145 cells

Figure S1

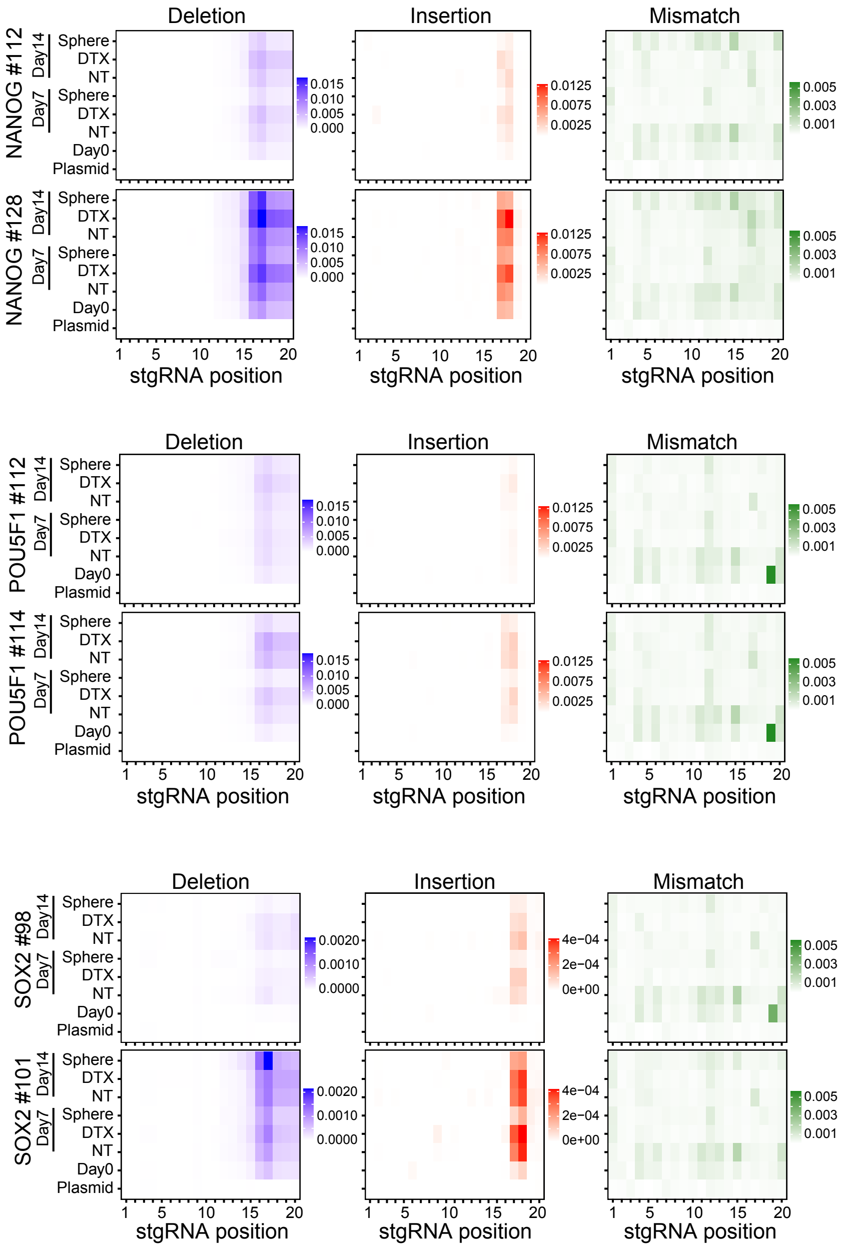
Deletion
Insertion
Mismatch
NANOG #112
NANOG #128
POU5F1 #112
POU5F1 #114
SOX2 #98
SOX2 #101
Day14
Day7
Sphere
DTX
NT
Day0
Plasmid
stgRNA position
1
5
10
15
20
0.015
0.010
0.005
0.000
0.0125
0.0075
0.0025
0.003
0.001
0.0020
0.0010
0.0000
4e−04
2e−04
0e+00

Figure S2

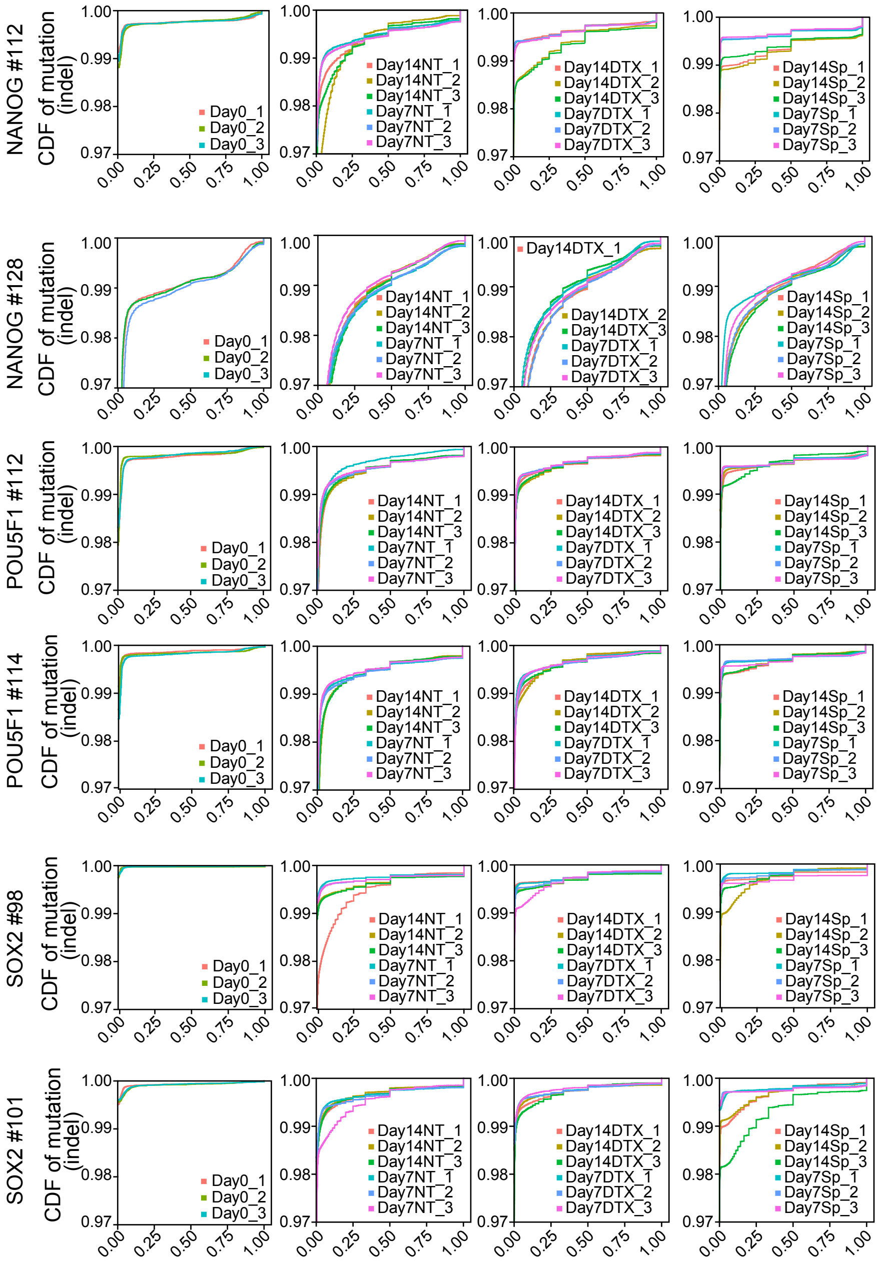

NANOG #112
NANOG #128
POU5F1 #112
POU5F1 #114
SOX2 #98
SOX2 #101
CDF of mutation (indel)
Day0_1
Day0_2
Day0_3
Day14NT_1
Day14NT_2
Day14NT_3
Day7NT_1
Day7NT_2
Day7NT_3
Day14DTX_1
Day14DTX_2
Day14DTX_3
Day7DTX_1
Day7DTX_2
Day7DTX_3
Day14Sp_1
Day14Sp_2
Day14Sp_3
Day7Sp_1
Day7Sp_2
Day7Sp_3

Figure S3

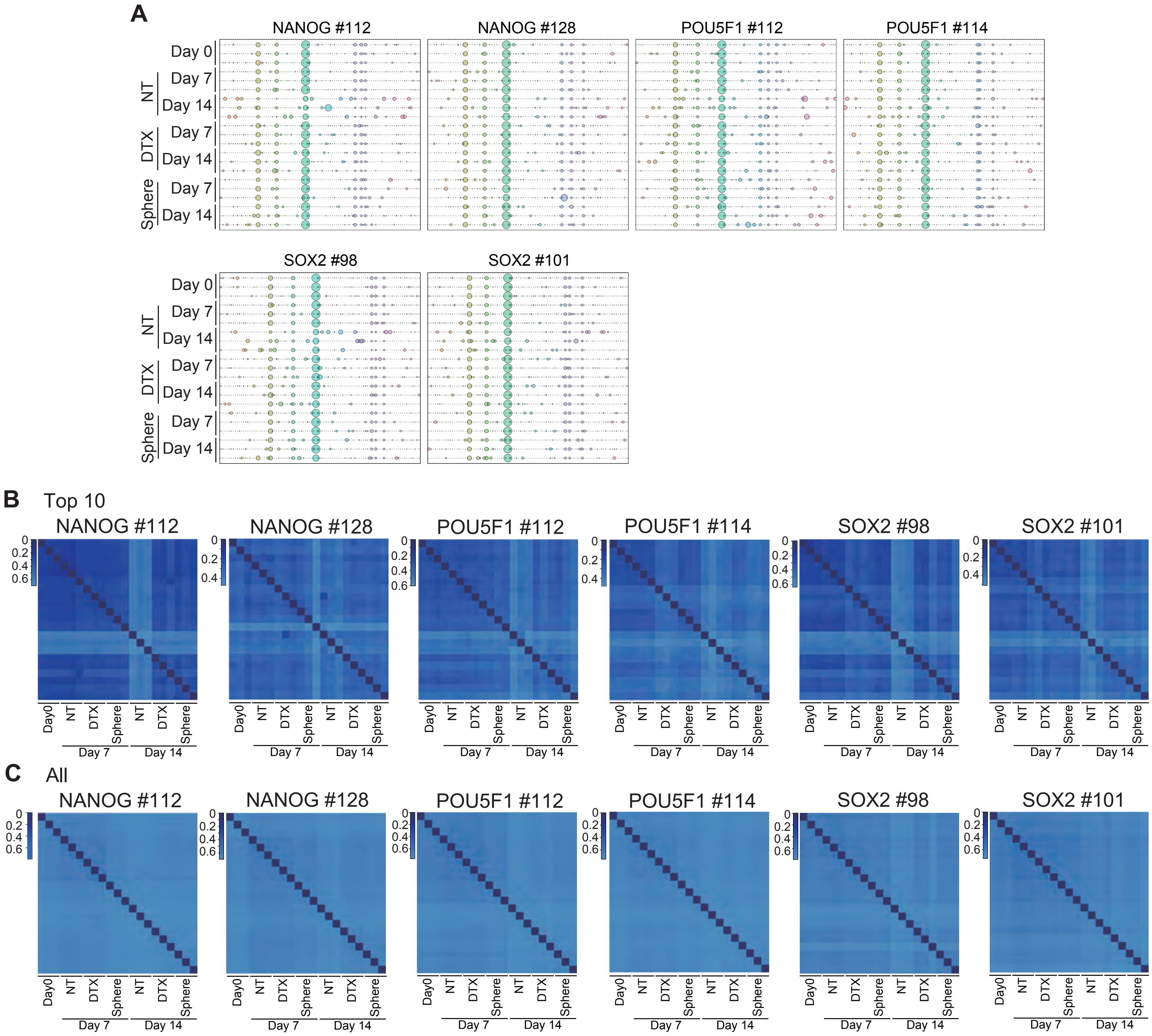
A
NANOG #112
NANOG #128
POU5F1 #112
POU5F1 #114
SOX2 #98
SOX2 #101
Day 0
NT
Day 7
Day 14
DTX
Day 7
Day 14
Sphere
Day 7
Day 14
B
Top 10
NANOG #112
NANOG #128
POU5F1 #112
POU5F1 #114
SOX2 #98
SOX2 #101
0
0.2
0.4
0.6
Day0
NT
DTX
Sphere
NT
DTX
Sphere
Day 7
Day 14
C
All
NANOG #112
NANOG #128
POU5F1 #112
POU5F1 #114
SOX2 #98
SOX2 #101
0
0.2
0.4
0.6
Day0
NT
DTX
Sphere
NT
DTX
Sphere
Day 7
Day 14

Figure S4

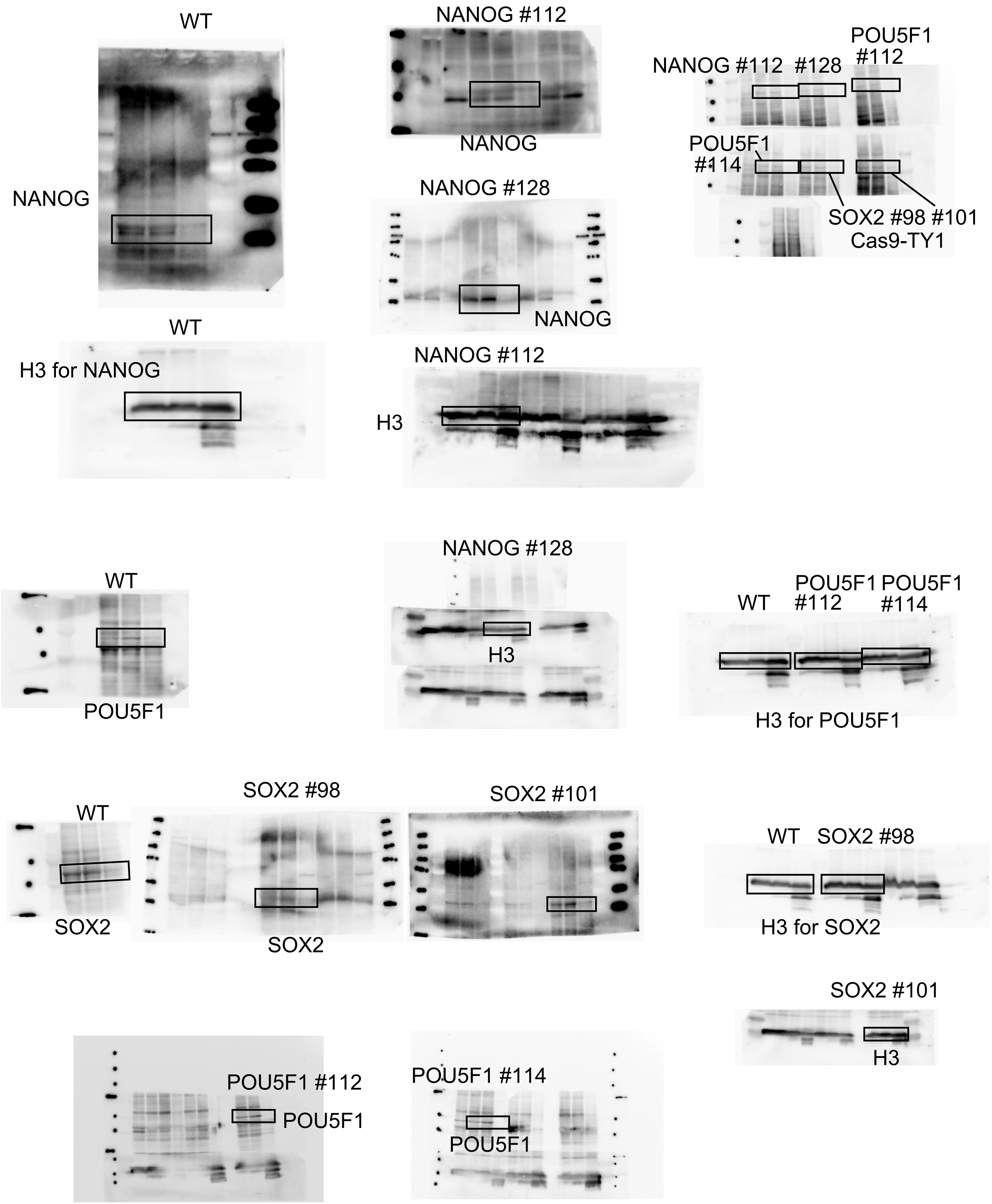

WT
NANOG
NANOG #112
NANOG
NANOG #112 #128
POU5F1 #112
POU5F1 #114
SOX2 #98 #101
Cas9-TY1
NANOG #128
NANOG
WT
H3 for NANOG
NANOG #112
H3
WT
POU5F1
NANOG #128
H3
WT
POU5F1 #112
POU5F1 #114
H3 for POU5F1
WT
SOX2
SOX2 #98
SOX2
SOX2 #101
WT
SOX2 #98
H3 for SOX2
SOX2 #101
H3
POU5F1 #112
POU5F1
POU5F1 #114
POU5F1

Table 1. The mean significant Spearman's correlations between mutation size of stgRNA and weighted number of cell barcode reads in DU145 cells

NANOG #112	NT	DTX	Sphere	total
Day 0	0.15084268	-	-	0.15084268
Day 7	0.21315188	0.25839764	0.18485206	0.21880053
Day 14	0.30707507	0.40959186	0.21077919	0.30914871
Day 7+Day 14	0.26011348	0.33399475	0.19781563	0.26397462

NANOG #128	NT	DTX	Sphere	total
Day 0	0.21243562	-	-	0.21243562
Day 7	0.29823256	0.34826646	0.29545658	0.3139852
Day 14	0.37051868	0.38781393	0.40101021	0.3864476
Day 7+Day 14	0.33437562	0.3680402	0.34823339	0.3502164

POU5F1 #112	NT	DTX	Sphere	total
Day 0	0.20116341	-	-	0.20116341
Day 7	0.22713807	0.30403789	0.21230744	0.2478278
Day 14	0.27527996	0.45500784	0.29374913	0.34134564
Day 7+Day 14	0.25120902	0.37952287	0.25302828	0.29458672

POU5F1 #114	NT	DTX	Sphere	total
Day 0	0.1767808	-	-	0.1767808
Day 7	0.25948499	0.29843192	0.19680559	0.25157417
Day 14	0.4000513	0.43264499	0.36335375	0.39868335
Day 7+Day 14	0.32976814	0.36553846	0.28007967	0.32512876

SOX2 #98	NT	DTX	Sphere	total
Day 0	0.13050151	-	-	0.13050151
Day 7	0.17763144	0.20721606	0.15236738	0.17907163
Day 14	0.24090878	0.34915232	0.25964547	0.28323552
Day 7+Day 14	0.20927011	0.27818419	0.20600643	0.23115358

SOX2 #101	NT	DTX	Sphere	total
Day 0	0.18075414	-	-	0.18075414
Day 7	0.21770528	0.21647747	0.17719019	0.20379098
Day 14	0.23513658	0.3699066	0.29745854	0.30083391
Day 7+Day 14	0.22642093	0.29319203	0.23732436	0.25231244
